# Respiratory tract infections in children with allergic asthma on allergen immunotherapy during influenza season

**DOI:** 10.1038/s41598-021-81558-0

**Published:** 2021-01-22

**Authors:** Yuyun Li, Dongming Wang, Lili Zhi, Yunmei Zhu, Lan Qiao, Yan Zhu, Xin Hu, Qian Wang, Yuan Cao, Yan Gao, Yousheng Chen, Zeng Zhang, Fangjie Bi, Guangxing Yan

**Affiliations:** 1 Zibo Central Hospital, No. 54, Gongqingtuan West Road, Zibo, 255036 Shandong Province People’s Republic of China; 2 People’s Hospital of Huantai County, No. 2198, Huantai Street, Zibo City, 256400 Shandong Province China; 3 Zibo Central Hospital, No. 54, Gongqingtuan West Road, Zibo, 255036 Shandong Province People’s Republic of China; 4 Zhangdian District Center for Disease Control and Prevention, No. 184, Xincun West Road, Zibo, 255000 Shandong Province China; 5 Respiratory Medicine Department, Zibo Central Hospital, No. 54, Gongqingtuan West Road, Zibo, 255036 Shandong Province People’s Republic of China; 6 Department of ENT, Zibo Central Hospital, No. 54, Gongqingtuan West Road, Zibo, 255036 Shandong Province People’s Republic of China; 7 Internal Cardiovascular Department, Zibo Central Hospital, No. 54, Gongqingtuan West Road, Zibo, 255036 Shandong Province People’s Republic of China; 8 Pediatrics, Zibo Municipal Hospital, No. 139, Huangong Road, Zibo, 255400 Shandong Province China; 9 Shandong Provincial Qianfoshan Hospital, The First Affiliated Hospital of Shandong First Medical University, Shandong Institute of Respiratory Diseases, Jinan, 250013 China

**Keywords:** Diseases, Medical research

## Abstract

To describle how respiratory tract infections (RTIs) that occurred in children with allergic asthma (AA) on allergen immunotherapy (AIT) during an influenza season. Data including clinical symptoms and treatment history of children (those with AA on AIT and their siblings under 14 years old), who suffered from RTIs during an influenza season (Dec 1st, 2019–Dec 31st, 2019), were collected (by face to face interview and medical records) and analyzed. Children on AIT were divided into 2 groups: stage 1 (dose increasing stage) and stage 2 (dose maintenance stage). Their siblings were enrolled as control. During the study period, 49 children with AA on AIT (33 patients in stage 1 and 16 patients in stage 2) as well as 49 children without AA ( their siblings ) were included. There were no significant differences in occurrences of RTIs among the three groups (*p* > 0.05). Compared with children in the other two groups, patients with RTIs in stage 2 had less duration of coughing and needed less medicine. Children on AIT with maintenance doses had fewer symptoms and recovered quickly when they were attacked by RTIs, which suggested that AIT with dose maintenance may enhance disease resistance of the body.

## Introduction

Allergic diseases have increased in children and adults with time^1–4^. Among them, allergic asthma (AA) is a chronic airway inflammation disease. It represents a significant health problem worldwide and is associated with much morbidity, mortality, and loss of productivity^5^, which has attracted much attention from patients and medical practitioners. The treatments of AA include avoidance of allergens, education of patients, medicines and allergen immunotherapy (AIT). And AIT is the only causal treatment of AA^6^. Patients can achieve AA well controlled in post-treatment follow-up over a recommended period of 3–4 years of AIT^7,8^. Then more and more patients with AA have accepted AIT, which should sustain for 3 years at least.

Respiratory tract infections (RTIs) are common for human beings in influenza seasons. For patients with AA, RTIs are very common causes of exacerbations of asthma, being implicated in 80% of exacerbations in children and 50% of exacerbations in adults^9–11^. The most commonly identified viruses associated with exacerbations of asthma are *rhinovirus*, *coronavirus*, *influenza virus*, *parainfluenza virus* and *respiratory syncytial virus*^9,12^, which are frequently identified in patients with RTIs in influenza seasons.

It is reported that AIT can enhance host defence against RTIs in *mouse* model with AA^13^. However, no reports have been found about how RTIs occur in children with AA on AIT. In our clinical practice, many children with AA sensitized to *house dust mites (HDM)* on AIT often suffered from RTIs. The aim of this study was to discuss how RTIs occurred in children with AA sensitized to *HDM* on AIT by face to face interview and medical records.

## Materials and methods

### Subjects

Children with AA sensitized to *HDM* under 14 years old, who were receiving AIT during Dec 1st and Dec 31st of the year 2019 and whose clinical symptoms were well controlled, were included in this research. At the same time, their siblings of the patients who didn’t suffer from allergic diseases were also enrolled in this research as control. The diagnosis of AA had to comply with the diagnostic criteria of AA according to the GINA guidelines^14^. All patients enrolled in this study had not received any influenza vaccines. The exclusion criteria were suspected cases of digestive tract infections, chronic RTIs such as tuberculosis, chronic respiratory diseases, immunosuppressive status (e.g., HIV infection, chemotherapy) and leukaemia. Patients with no siblings, or with siblings suffering from allergic diseases, or with siblings under 5 years old, or with siblings older than 14 years old, or accepting any influenza vaccines, or with rhinitis, or with bad controlled asthmas, and or refusing the research were also excluded from this research.

### Ethics statement

The study was conducted in accordance with the Good Clinical Practice (GCP) guidelines. This study was approved by the Institutional Review Board of Zibo Central Hospital according to the ethical principles that have their origins in the Declaration of Helsinki and its subsequent amendments. Written informed consents were obtained from guardians of children.

### Data collection

Data including age, gender, medical records of AIT, flu vaccination history, RTIs attack, coughing, fever, sore throat, wheezing, chest tightness, nasal congestion, sneezing, nasal itching, runny nose, medicine for RTIs, hospitalization, fluid therapy, other symptoms of RTIs (e.g. headache, vomiting) and seeking medical help were collected from children with AA sensitized to *HDM* who were receiving AIT and their siblings without allergic diseases by a face to face interview and medical records. Presence or absence of RTIs was determined by the health professionals according to symptoms and medical records. The courses of AIT treatment were acquired from medical records of the children with AA. AIT consisted of dose increasing treatment (stage 1) and dose maintenance treatment (stage 2). The number of Children with RTIs who visited paediatric clinic of Zibo Central Hospital and the number of those who were hospitalized from Jan 1st to Dec 31st of the year 2019 were also collected.

### Statistical analysis

All statistical analyses were performed with SPSS software, version 21.0 (IBM Corp, Armonk, NY, USA). Continuous variables were expressed as mean ± standard deviation. The method of t-test was used to compare means of 2 groups while ANOVA were used to compare means of multiple groups. LSD test were used for the inter-comparison of 2 means in the multiple groups. Categorical variables were assessed by the Chi-square test. *p* < 0.05 was indicated as a statistically significant difference.

## Results

### Subjects

**Figure 1:**
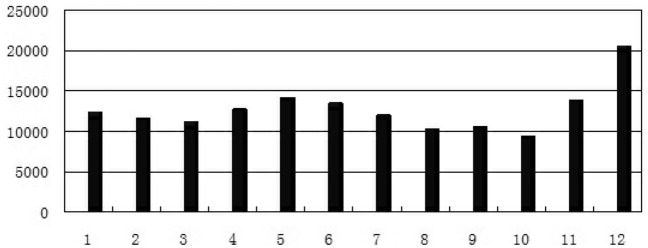
The numbers of outpatients with RTIs in months of the year 2019.

**Figure 2:**
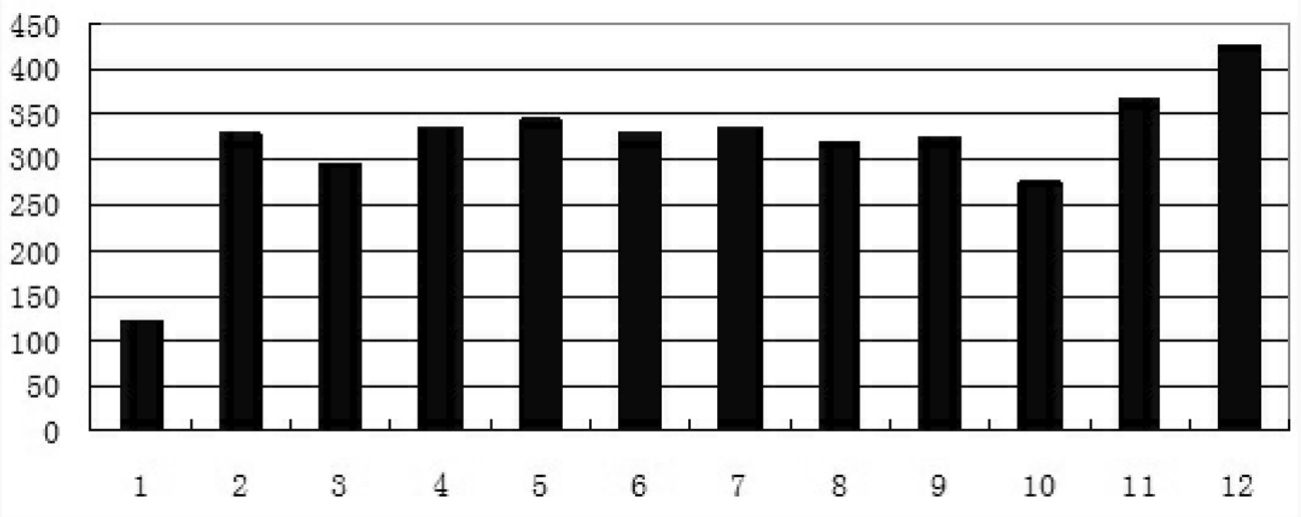
The numbers of children with RTIs hospitalized in months of the year 2019.

From Jan 1st to Dec 31st of the year 2019, there were 152,440 children in total who visited paediatrics of Zibo Central Hospital for RTIs. In December of 2019, there were 20,540 children outpatients with RTIs and 425 children inpatients with RTIs, which were much higher than those in any other month of the year (Figs. 1, 2). And during the study period, there were 353 children with AA sensitized to *HDM* on SCIT in the allergy clinic. Among them, 304 children with AA sensitized to *HDM* were excluded from this research for no siblings (109 cases), accepting influenza vaccines (49 cases), siblings with allergic diseases (42 cases), siblings under 5 years old (33 cases), siblings older than 14 years old (26 cases), rhinitis (22 cases), bad controlled asthmas (18 cases), and refusing the research (5 cases). Then 49 children with AA sensitized to *HDM* who were receiving SCIT as well as their siblings (49 children) were included in this research. Among them, 33 children with AA sensitized to *HDM* were receiving dose increasing treatment of SCIT (stage 1) while 16 children with AA sensitized to *HDM* were receiving dose maintenance treatment of SCIT (stage 2). Their siblings were also enrolled in this research as control. There were no significant differences in ages (F = 0.12, *p* > 0.05) and genders (χ^2^ = 0.05, *p* > 0.05) among the 3 groups.

### RTIs in children with AA on SCIT and control

During the study period, there were 21 cases, 7 cases and 32 cases with RTIs occurred in stage1, stage 2 and control, respectively. There were no significant differences in occurrences of RTIs among the 3 groups (*p* > 0.05). The occurrences of lower RTIs, fever, high fever, coughing, wheezing, medicine, seeking medical help, recurrent infections, fluid therapy and hospitalization were lower in stage 2 than those in stage 1 and control, respectively. However, no significant differences were observed in these indicators among the 3 groups (*p* > 0.05). (Table 1).

**Table1:** RTIs in children with AA on SCIT and control.

	Stage 1	Stage 2	Control	Statistical	p
n	33	16	49		
Age (years )	7.88 ± 2.57	8.13 ± 2.68	7.80 ± 2.07	F = 0.12	*p* > 0.05
Gender(m:f)	15:18	7:9	23:26	χ2 = 0.05	*p* > 0.05
**Infection**					
Upper RTIs	16	7	24		
Lower RTIs	5	0	8	χ2 = 4.15	*p* > 0.05
Fever	12	3	17	χ2 = 1.71	*p* > 0.05
High fever	3	0	6	χ^2^ = 2.17	*p* > 0.05
coughing	15	6	26	χ^2^ = 1.30	*p* > 0.05
wheezing	2	0	5	χ^2^ = 1.98	*p* > 0.05
Chest tightness	0	0	4	χ^2^ = 4.17	*p* > 0.05
Nasal congestion	5	3	5	χ^2^ = 0.92	*p* > 0.05
Sneezing	2	0	5	χ^2^ = 1.98	*p* > 0.05
Nasal itching	1	0	0	χ^2^ = 1.99	*p* > 0.05
Runny nose	5	4	19	χ^2^ = 5.51	*p* > 0.05
Sore throat	16	4	13	χ^2^ = 4.90	*p* > 0.05
Headache	2	1	1	χ^2^ = 1.04	*p* > 0.05
Other symptoms	4	1	5	χ^2^ = 0.41	*p* > 0.05
Medicine	18	7	23	χ^2^ = 0.67	*p* > 0.05
Seeking medical advice	8	1	11	χ^2^ = 2.40	*p* > 0.05
Recurrent infections	2	0	3	χ^2^ = 1.03	*p* > 0.05
Fluid therapy	6	0	9	χ^2^ = 3.57	*p* > 0.05
Hospitalization	4	0	8	χ^2^ = 2.99	*p* > 0.05

### Clinical characteristics of children with RTIs

In order to discuss the severity of the RTIs, children with RTIs were divided into 3 groups: stage1, stage 2 and control, respectively. Duration of coughing was significantly less in stage 2 than those in stage 1 and control (*p* < 0.05), respectively. But no significant difference was observed in duration of coughing between stage 1 and control (*p* > 0.05). It was suggested that children with RTIs in stage 2 were susceptible to suffer less duration of coughing. The duration of medicine used for RTIs in stage 2 was less than that in stage 1 and control respectively, but there were no significant differences among them (*p* > 0.05). No seeking medical help, recurrent infections, fluid therapy and hospitalization occurred in stage 2, which suggested that patients with dose maintenance treatment may have mild symptoms during influenza season. More frequent seeking medical help were found in stage 1 than in stage 2 and control (*p* < 0.05), respectively. (Table 2).

**Table2:** Clinical characteristics of children with RTIs.

	Stage 1	Stage 2	control	Statistical analysis	p
n	21	7	32		
Age (years )	7.97 ± 2.56	7.86 ± 2.04	7.13 ± 2.1.72	F = 1.14	*p* > 0.05
Gender(m:f)	10:11	3:4	17:15	χ^2^ = 0.32	*p* > 0.05
**Infection**					
Upper RTIs	16	7	24		
Lower RTIs	5	0	8	χ^2^ = 2.20	*p* > 0.05
Fever	12	3	17	χ^2^ = 0.43	*p* > 0.05
High fever	3	0	6	χ^2^ = 1.60	*p* > 0.05
Coughing	15	6	26	χ^2^ = 0.98	*p* > 0.05
Duration of coughing	6.27 ± 2.79^a^	3.33 ± 1.86	6.50 ± 2.57^b^	F = 3.80	*p* < 0.05
Wheezing	2	0	5	χ^2^ = 1.51	*p* > 0.05
Chest tightness	0	0	4	χ^2^ = 3.75	*p* > 0.05
Nasal congestion	5	3	5	χ^2^ = 2.60	*p* > 0.05
sneezing	2	0	5	χ^2^ = 1.51	*p* > 0.05
Nasal itching	1	0	0	χ^2^ = 2.48	*p* > 0.05
Runny nose	5	4	19	χ^2^ = 3.50	*p* > 0.05
Sore throat	16	4	13	χ^2^ = 6.50	*p* < 0.05
Headache	2	1	1	χ^2^ = 1.57	*p* > 0.05
Other symptoms	4	1	5	χ^2^ = 0.14	*p* > 0.05
Medicine	18	7	23	χ^2^ = 3.50	*p* > 0.05
Duration of medicine	5.50 ± 3.09	3.70 ± 1.98	5.57 ± 2.95	F = 1.20	*p* > 0.05
Seeking medical advice	8	1	2	χ^2^ = 8.68	*p* < 0.05
Recurrent infections	2	0	3	χ^2^ = 0.72	*p* > 0.05
Fluid therapy	6	0	9	χ^2^ = 2.64	*p* > 0.05
Furation of fluid therapy	7.50 ± 1.22		6.44 ± 1.13	t = 1.73	*p* > 0.05
Hospitalization	4	0	8	χ^2^ = 2.26	*p* > 0.05
Duration of hospitalization	5.75 ± 3.20		6.50 ± 1.93	t = 0.51	*p* > 0.05

^a^Comparison between stage1 and stage2, *p* < 0.05; ^b^Comparison between stage2 and control, *p* < 0.05.

## Discussions

AA is a multi-factorial disease of the airway that precipitates from genetic predisposition and environmental triggers. The World Health Organization estimates that 235 million people have asthma and an additional 100 million people will develop the disease over the next 15 years^15,16^. Patients with AA are susceptible to suffer from RTIs due to an impaired antimicrobial defence^17–20^. A prospective study during the 2009 influenza pandemic showed that H1N1 preferentially infected asthmatics more than non-asthmatics^21^. AA exacerbations could be caused by RTIs frequently^22^. However, subsequent studies noted that asthmatics had fewer severe outcomes (including reduced bacterial pneumonia) compared to non-asthmatics^23–27^, which may be explained by the accelerated clearance of viruses in patients with AA^15,28^. But it was regretful that we could get few children with AA qualified who didn’t receive AIT in allergy clinic. So we could not acquire medical data of RTIs in this kind of patients for comparison during the study period in this research.

RTIs including common cold, acute tonsillitis, acute rhinosinusitis, flu-like illness, acute bronckitis, and pneumonia are the most common diseases in human beings^29^. Typical symptoms of patients with RTIs include sneezing, nasal congestion and discharge, sore throat, cough, fever, headache, and malaise, which were similar to those of patients with allergic rhinitis and asthma. RTIs are usually diagnosed clinically, based on symptoms. In this research, fever or sore throat was necessary in the diagnosis of RTIs. RTIs consist of upper RTIs and lower RTIs. Compared with upper RTIs, lower RTIs such as pneumonia is considered more severe. Most RTIs occur in winter days in northern hemisphere^30^. In our research, RTIs outbreak occurred in December, which suggested an influenza season. During that time, many patients with AA who were receiving AITs also suffered from RTIs. In order to discuss how RTIs occurred in children with AA who were receiving AIT, the research on RTIs in children with AA sensitized to *HDM* on AIT as well as their siblings in December of 2019 were conducted.

In the research, the occurrences of RTIs in patients with AA were similar to those in patients without AA, which was not consistent with the previous report^21^. And the occurrences of lower RTIs, fever, high fever, coughing, wheezing, medicine, seeking medical help, recurrent infections, fluid therapy and hospitalization in children with AA receiving dose maintenance treatment were less than those in patients with AA receiving dose increasing treatment and in patients without AA, which suggested that AIT with dose maintenance treatment may enhance RTIs resistance of the body. It was difficult to explain the phenomenon and further study should be needed.

Frequent seeking medical help, recurrent infections, fluid therapy and hospitalization were important indicators in evaluating the severity of RTIs. In our research, no seeking medical help, recurrent infections, fluid therapy and hospitalization occurred in children with AA on dose maintenance SCIT. Especially, the duration of coughing in children with AA on dose maintenance SCIT was significantly less than those in children with AA on dose increasing SCIT and their siblings. All those above suggested that children with AA on dose maintenance SCIT were prone to have mild symptoms after RTIs attack. The explanation may be that SCIT can play important role in the protection of children from RTIs, which was consistent with the previous results^31^. Frequent seeking medical help was very high in children with AA on dose increasing SCIT. For guardians of the children with AA often worried about the health of children. They couldn’t stop visiting doctors for medical help at the beginning of SCIT. The occurrence of RTIs in children with AA on dose increasing SCIT was similar to that in children without AA, which suggested that SCIT at dose increasing treatment could not protect the children from RTIs attacks.

However, this research still had some limitations. First, the number of the patients was limited.. And many variables couldn’t get statistical significances. Second, limited to our research equipment, we couldn’t detect the microbes of the RITs in laboratory. The influenza season was judged by the number of children with RTIs visiting our hospital and clinical experiences. Third, it was a retrospective research. Many data couldn’t be acquired. Fourth, almost all the patients who came to allergy clinic for SCIT, no enough children with AA qualified who didn’t accept SCIT could be acquired for comparison. Fourth, diagnoses were based on clinical symptoms; however, that is a rule in general practice^29^.

In summary, Children on AIT with maintenance dose had fewer symptoms and recovered quickly when they were attacked by RTIs, which suggested that AIT with dose maintenance may enhance disease resistance of the body.
